# The influence of mesenchymal stem cells on the severity of hypoxic-ischemic brain damage in neonatal rats via serum cytokines

**DOI:** 10.1016/j.ibneur.2025.12.002

**Published:** 2025-12-05

**Authors:** Sifeng Yue, Feng Lin, Zenghong Huang, Xinyi Liang, Shuchun Lü, Yuan Tan

**Affiliations:** a Neonatology Department, The First Affiliated Hospital of Guilin Medical University, Guilin, Guangxi 541000, China; b Guilin Medical University, Guilin, Guangxi 541000, China

**Keywords:** Mesenchymal stem cells, Serum cytokine, Hypoxic-ischemic brain damage, Repair

## Abstract

**Background:**

Neonatal hypoxic-ischemic brain damage (Hypoxic‑Ischemic Brain Damage, HIBD) is a severe neurological disorder caused by perinatal asphyxia. Its severe long-term sequelae impose a heavy burden on families and society. Therefore, effective early diagnosis and intervention for HIBD are crucial for improving prognosis.

**Objective:**

To investigate the correlation between serum levels of interleukin-6 (IL-6), interleukin-18 (IL-18), and tumor necrosis factor-α (TNF-α) and the severity of hypoxic-ischemic brain damage (HIBD) in neonatal SD rats, as well as the effects of mesenchymal stem cells (MSCs) on serum cytokine levels and the repair of HIBD in rats.

**Methods:**

250 7-day-old SD rats were selected and a HIBD model was established using the Rice-Vannucci method. The rats were randomly divided into 5 groups: 1) control group; 2) mild HIBD group; 3) severe HIBD group; 4) mild HIBD + MSCs group; 5) severe HIBD + MSCs group. The MSCs intervention group rats were injected with MSCs into the lumbar spine 1 and 8 days after modeling. Longa scores were evaluated at 0, 3, 7, 11, and 15 days after modeling, and serum levels of IL-6, IL-18, and TNF-α were detected. Brain tissue was stained with hematoxylin-eosin (HE) for Longa scoring. The correlation between serum cytokines and the severity of brain injury was analyzed, and the regulatory effect of MSCs intervention on cytokine expression levels and its role in neural injury repair were further explored.

**Results:**

(1) The Longa scores and serum levels of IL-6, IL-18, and TNF-α in the mild HIBD group and severe HIBD group were significantly higher than those in the control group at all stages (P < 0.05), and the severe HIBD group had significantly higher levels than the mild HIBD group at all stages (P < 0.05). (2) After treatment, HE staining showed that the brain tissue damage in the mild HIBD group and mild HIBD + MSCs group improved, and the Longa scores and serum levels of IL-6, IL-18, and TNF-α decreased significantly at all time points (D3, D7, D11, D15) after treatment compared to those before treatment (D0). The Longa scores and serum levels of IL-6, IL-18, and TNF-α in the mild HIBD + MSCs group and severe HIBD + MSCs group decreased significantly at each time point (D3, D7, D11, D15) after treatment compared to those before treatment (D0).

**Conclusion:**

The serum levels of IL-6, IL-18, and TNF-α in rats are positively correlated with the severity of HIBD and can reflect the degree of brain tissue injury. They can be used for disease assessment; mesenchymal stem cell treatment can significantly reduce the serum levels of IL-6, IL-18, and TNF-α in HIBD rats, alleviate inflammatory responses, improve symptoms, and repair brain injury.

## 1 Introduction

Neonatal hypoxic‑ischemic brain damage (Hypoxic‑Ischemic Brain Damage, HIBD) refers to a neurological disorder in newborns caused by asphyxia‑induced cerebral blood flow disturbances during the perinatal period. It often leads to severe sequelae, imposing a heavy psychological and economic burden on affected families. Effective prevention, early diagnosis, and timely treatment are therefore crucial for improving neurological function and reducing long‑term complications in these infants ^(^Shao et al., 2019^)^.

The pathological mechanisms of HIBD are highly complex (Guo and Yuan, 2021, Xiao et al., 2021). Under hypoxic‑ischemic conditions, brain tissue and neural cells trigger excessive activation of inflammatory cytokines, initiating a cascade of inflammatory responses and promoting the release of neurotoxic mediators. This inflammatory cascade has become a core pathological pathway leading to neonatal neuronal injury (Zhou and Lu, 2021). Mesenchymal stem cells (MSCs), owing to their immunomodulatory, anti‑inflammatory, and tissue‑regenerative properties, have demonstrated significant neuroprotective effects in ischemic brain injury models by suppressing inflammation and promoting reconstruction of neural structures at injury sites (Duan and Liang, 2025, Ding et al., 2024).

In the present study, we employed a controlled experimental design in which HIBD model rats served as the experimental groups and healthy littermates served as controls. We systematically measured serum levels of key inflammatory cytokines—interleukin‑6 (IL‑6), interleukin‑18 (IL‑18), and tumor necrosis factor‑α (TNF‑α)—using enzyme‑linked immunosorbent assay (ELISA). We analyzed the correlation between serum cytokine profiles and the severity of brain injury, and further investigated how MSC intervention modulates cytokine expression and contributes to neural repair. Our aim was to provide a scientific basis for clinical assessment of HIBD severity and to optimize MSC‑based therapeutic strategies for neonatal hypoxic‑ischemic brain injury.

## 2 Materials and methods

### 2.1 Experimental animals

Seven‑ to eight‑week‑old Sprague–Dawley (SD) rats were supplied by Jiangsu Huachuang Xinnuo Pharmaceutical Technology Co., Ltd. (License No. SYXK [Su] 2020‑0041). All parental rats were acclimated and bred in the Experimental Animal Center of Guilin Medical University, yielding second‑generation pups for the study. Animals were housed under standard conditions: temperature 24 ± 1 ℃, humidity 50 ± 5 %, 12 h light/12 h dark cycle, with ad libitum access to specialized feed; pregnant females received breeder feed to ensure sufficient nutrition. The animal protocol was approved by the Ethics Committee of Guilin Medical University (Approval No. GLMC202405281) and conformed to the Regulations on the Administration of Laboratory Animals and the 3 R principles (Replacement, Reduction, Refinement).

### 2.2 Methods

#### 2.2.1 Establishment of the HIBD model and grouping

A total of 250 seven‑day‑old SD pups (12–20 g, both sexes) were randomly assigned to five groups (n = 50 each): 1. Control 2. Mild HIBD 3. Severe HIBD 4. Mild HIBD + MSCs 5. Severe HIBD + MSCs. The Rice–Vannucci method was used to induce HIBD. Under ether anesthesia, the neck was disinfected with 75 % alcohol and a midline incision was made to expose the right common carotid artery, which was then doubly ligated and transected. The incision was sutured and disinfected. After a 0.5–1 h recovery with the dam, pups were placed in a 37 ℃ hypoxia chamber perfused with 8 % O₂/92 % N₂. The mild HIBD group underwent 2 h of hypoxia; the severe HIBD group underwent 4 h. Thereafter, pups were returned to the dam. Control pups underwent the same surgery without ligation or hypoxia. MSC intervention groups received intrathecal lumbar injections of MSCs which is from SD rat bone marrow on days 1 and 8 post–modeling, each rat was injected with 5 × 10^5–1 × 10^6 stem cells each time(The MSCs were purchased from Cyagen Biosciences (Guangzhou) Inc. (Item No.: RASMX-01001; Batch No.: 210408U31). The cells were derived from a female Sprague-Dawley rat within one week after birth and were cryopreserved at passage 2. All quality control tests of the product were qualified.); all other groups received equal volumes of saline at the same time points.

#### 2.2.2 Model confirmation criteria

Following surgery, the four HIBD groups were assessed for neurological deficits, including impaired motor coordination, reduced muscle tone, balance dysfunction, loss or limitation of righting reflex, and absence of pain response to tail pinch. Animals exhibiting any of these signs were deemed successfully modeled; those without such features were excluded.

#### 2.2.3 Neurological function assessment

Neurological function was assessed on days 0, 3, 7, 11, and 15 after modeling (denoted as D0, D3, D7, D11, and D15). At each time point, ten rats from each group were randomly selected and evaluated using the Longa scoring method ^(^Longa et al., 1989^)^. The specific criteria were as follows: a score of 0 indicated no neurological deficit, with the rat exhibiting normal activity and symmetrical limb movement; a score of 1 reflected mild impairment, characterized by flexion of the left forelimb and slight circling toward the paralyzed side while walking; a score of 2 denoted moderate impairment, where the rat walked in circles toward the paralyzed side and showed impaired balance but retained spontaneous movement; a score of 3 represented severe damage, with the body leaning toward the paralyzed side and markedly reduced voluntary activity; and a score of 4 indicated extremely severe damage, manifesting as complete loss of spontaneous movement along with impaired consciousness ^(^Zhang et al., 2017^)^.

#### 2.2.4 Sample collection

1）On days 0, 3, 7, 11, and 15, ten rats per group were anesthetized and 0.3–0.5 ml of blood was drawn from the jugular vein. Serum was separated for cytokine assays. 2）Following blood collection, the same rats were euthanized under ether anesthesia and perfused transcardially with saline via the left ventricle, followed by fixation with 4 % paraformaldehyde until rigidity of the neck and limbs. Brains were harvested and post‑fixed in 4 % paraformaldehyde for 24 h, then processed for paraffin embedding and HE staining.

#### 2.2.5 Serum IL‑6, IL‑18, and TNF‑α measurement

The levels of IL-6, IL-18 and TNF-α in the serum were quantitatively detected using the ELISA kit (RUIXIN BIOTECH). Briefly, samples and standards were added to microplates, incubated with detection antibodies at 37 ℃ in the dark for 60 min, washed, and developed. Optical density (OD) values were measured on a microplate reader. Each sample was assayed in duplicate. A four‑parameter logistic (4‑PL) standard curve (R² ≥ 0.99) was generated from seven standards (including zero) to calculate cytokine concentrations from sample OD values.

#### 2.2.6 Histopathological examination of brain tissue

Fixed brain tissues of neonatal rats were subjected to dehydration through a graded ethanol series (70 %, 80 %, 90 %, 95 %, and 100 %), followed by clearing with xylene. The tissues were then infiltrated with paraffin at 60–65°C, embedded in molds filled with molten paraffin, and placed in a cool, dry environment for solidification. The embedded paraffin blocks were sectioned, and the slices were dewaxed twice in xylene, then rehydrated through a descending ethanol series (100 %, 100 %, 95 %, 90 %, 80 %, and 70 %), with each step lasting five minutes. The sections were rinsed with distilled water, stained with hematoxylin and eosin (HE), dehydrated, cleared again, and finally mounted. After air drying at room temperature, the sections were examined under a microscope. Pathological changes and the extent of damage in the brain tissue of neonatal rats were observed and photographed under 400 × magnification.

### 2.3 Statistical analysis

Statistical analysis was performed using SPSS version 27.0. For data conforming to a normal distribution and homogeneity of variance, results were expressed as mean ± standard deviation (‾x ± SD). Two-way analysis of variance (ANOVA) was used for multi-group comparisons, and the least significant difference (LSD) method was applied for pairwise comparisons. Independent sample *t*-tests were used for intergroup comparisons. Statistical significance has been corrected using the Bonferroni method. For data with a skewed distribution, results were presented as median (lower quartile, upper quartile), and the Mann–Whitney *U* test was employed for comparisons between groups, while the Wilcoxon signed-rank test was used for within-group comparisons.

## 3 Results

### 3.1 HE staining results of brain tissue in neonatal SD rats

**Fig. 1:**
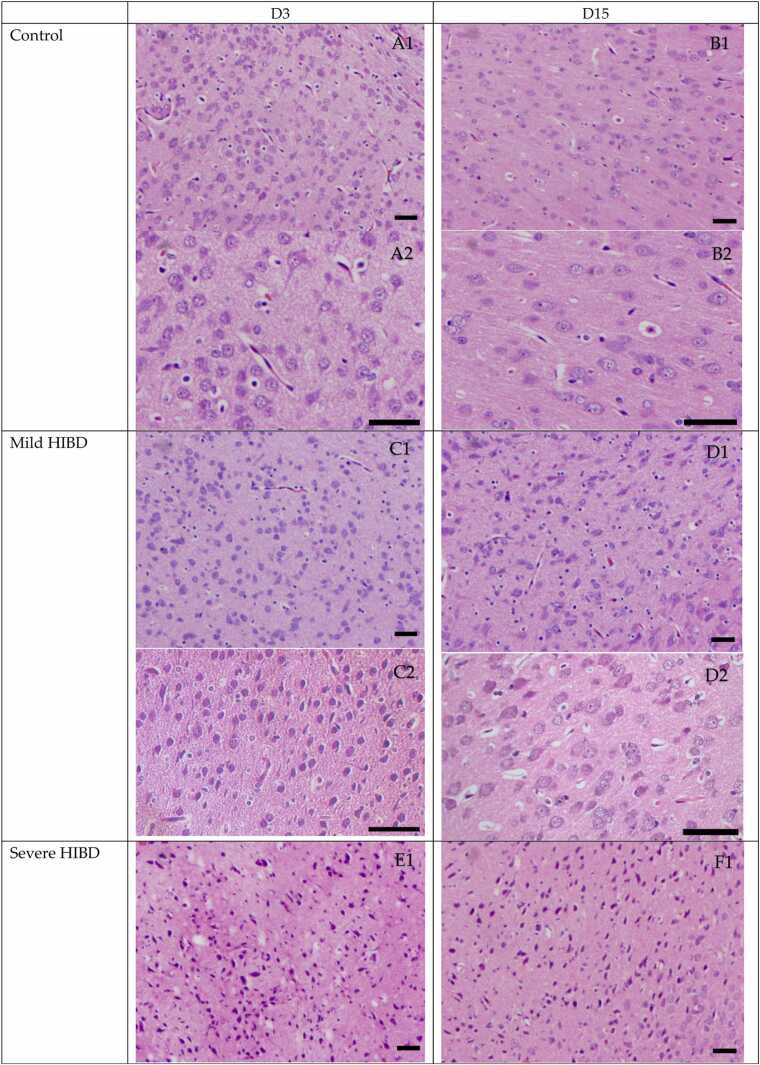

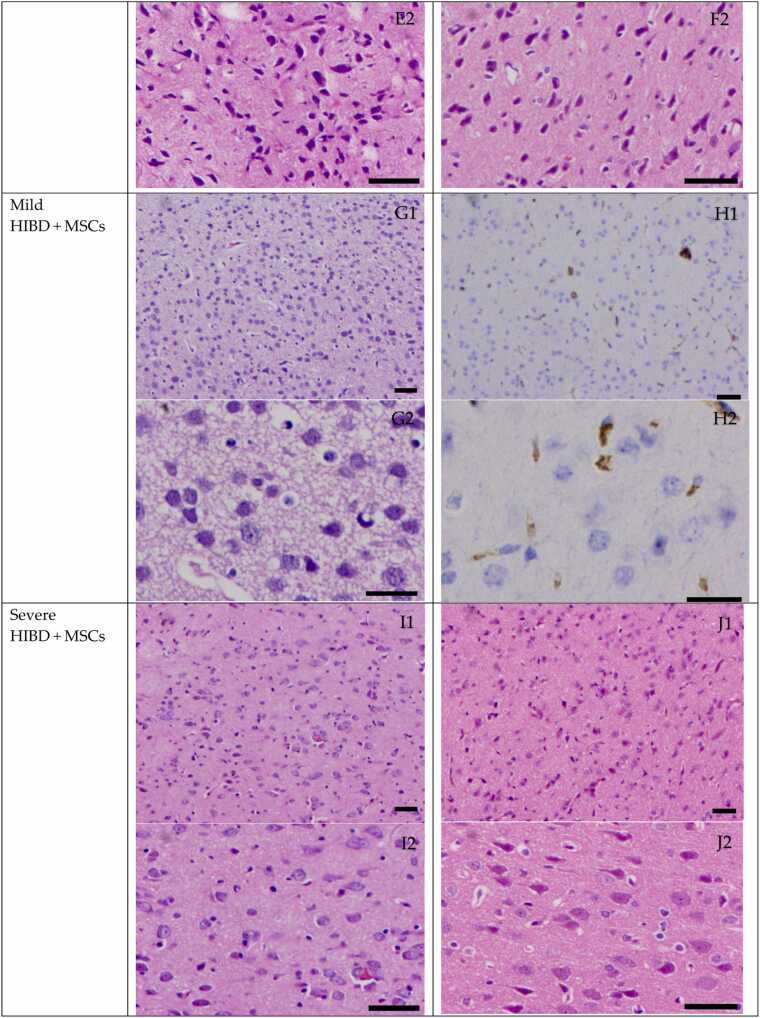
HE staining of paraffin section of brain tissue in SD rats (Panels A1, B1, C1, D1, E1, F1, G1, H1, I1 and J1 were imaged at a magnification of ×100, while panels A2, B2, C2, D2, E2, F2, G2, H2, I2 and J2 were imaged at ×400. Scale bars:50μm).

In the control group, neurons in the brain tissue of neonatal rats appeared intact, with normal morphology of the cytoplasm, nuclei, and nucleoli. In the mild HIBD and severe HIBD groups, neuronal damage was evident, with signs of degeneration and nuclear pyknosis, which were more pronounced in the severe HIBD group compared to the mild HIBD group (P < 0.05). After mesenchymal stem cell (MSC) treatment, both the mild HIBD + MSCs group and the severe HIBD + MSCs group showed marked improvement in neuronal injury on days 3 and 15 (P < 0.05). Signs of degeneration and nuclear pyknosis were significantly reduced. (See Fig. 1 and Table 1, Table 2)

**Fig. 2:**
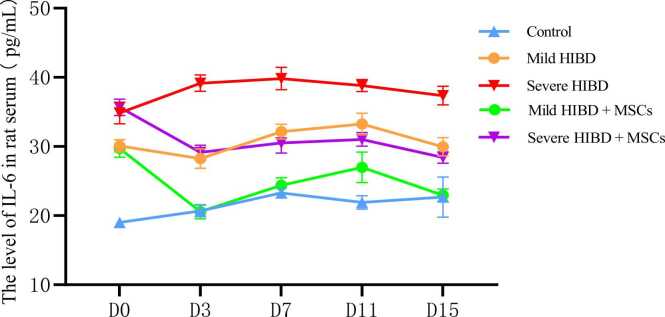
The level of IL-6 in rat serum.

**Fig. 3:**
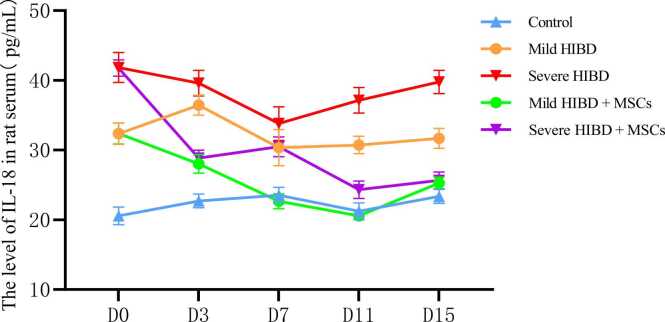
The level of IL-18 in rat serum.

**Fig. 4:**
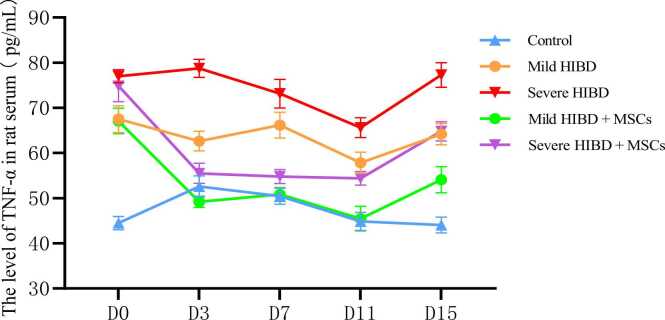
The level of TNF-α in rat serum.

**Table 1:** The ratio of shrunken cells to all cells in the HE staining images.

	Control （*n* = 5）	Mild HIBD（*n* = 5）	Severe HIBD（*n* = 5）	Mild HIBD + MSCs（*n* = 5）	Severe HIBD + MSCs（*n* = 5）
D3	0.052 ± 0.007	0.112 ± 0.027	0.177 ± 0.018	0.059 ± 0.015	0.103 ± 0.018
D15	0.069 ± 0.007	0.129 ± 0.037	0.192 ± 0.010	0.071 ± 0.008	0.142 ± 0.023

**Table 2:** Comparison of the ratio of shrunken cells to all cells in each group of rats.

	**T-value**		
	**ALL**	**D3**	**D15**
Control vs. mild HIBD	5.708*	4.872*	3.614*
Control vs. severe HIBD	20.257*	14.745*	21.853*
Mild HIBD vs. severe HIBD	5.683*	4.456*	3.679*
Mild HIBD vs. mild HIBD + MSCs	5.162*	3.842*	3.477*
Severe HIBD vs. severe HIBD + MSCs	6.026*	6.602*	4.407*

Note: “*” indicates P < 0.05

### 3.2 Clinical manifestations and neurological function assessment in each Group

During the modeling process of this experiment, rats exhibited mild cyanosis around the nose and perioral region within 1–2 min of hypoxia exposure, accompanied by increased respiratory rate, heightened activity, and subtle head tremors. As the hypoxia duration reached 4–5 min, these symptoms significantly worsened, with the animals displaying balance dysfunction and marked difficulty in autonomously righting themselves. After 1 h of sustained hypoxia, the rats gradually entered a suppressed state, with reduced intermittent agitation and a notable decline in overall activity levels.

Longa scores were analyzed for each group:

According to the Wilcoxon signed-rank test, significant differences were observed between D0 and all subsequent time points (D3, D7, D11, and D15) in both the mild HIBD + MSCs and severe HIBD + MSCs groups (P < 0.05), indicating statistical significance. (See Table 3)

**Table 3:** Analysis of Longa scores in each group of rats.

	Control （*n* = 10）	Mild HIBD（*n* = 10）	Severe HIBD（*n* = 10）	Mild HIBD + MSCs（*n* = 10）	Severe HIBD + MSCs（*n* = 10）
D0	0.0（0.0,0.3）	2.0（2.0,3.0）	3.0（2.8,3.3）	2.0（1.8,3.0）	3.0（2.8,3.3）
D3	0.0（0.0,0.0）	2.0（2.0,3.0）	3.0（3.0,3.0）	1.0（0.0,2.0）	2.0（1.8,3.0）
D7	0.0（0.0,0.0）	2.0（1.0,2.3）	3.0（2.0,3.0）	1.0（0.0,2.0）	2.0（1.0,2.0）
D11	0.0（0.0,0.0）	2.0（1.0,2.0）	2.0（2.0,3.0）	0.0（0.0,1.0）	2.0（1.0,2.0）
D15	0.0（0.0,0.0）	2.0（1.0,2.0）	2.0（2.0,3.0）	0.0（0.0,0.3）	1.5（1.0,2.0）

Note: In both the mild HIBD + MSCs group and the severe HIBD + MSCs group, comparisons of D0 vs D3, D0 vs D7, D0 vs D11, and D0 vs D15 all showed P < 0.05.

Based on the Mann–Whitney *U* test, there were statistically significant differences between the following group comparisons: control vs. mild HIBD, control vs. severe HIBD, mild HIBD vs. severe HIBD, mild HIBD vs. mild HIBD + MSCs, and severe HIBD vs. severe HIBD + MSCs (P < 0.001). However, when comparing mild HIBD vs. mild HIBD + MSCs and severe HIBD vs. severe HIBD + MSCs at each time point, no significant difference was observed at D0 (P > 0.05). (See Table 4)

**Table 4:** Comparison of Longa scores among groups of rats.

	Z					
	ALL	D0	D3	D7	D11	D15
Control vs. mild HIBD	9.032^**^	3.88^**^	4.038^**^	3.894^**^	4.119^**^	4.119^**^
Control vs. severe HIBD	9.152^**^	3.966^**^	4.108^**^	4.013^**^	4.081^**^	4.108^**^
Mild HIBD vs. severe HIBD	4.505^**^	2.071*	2.368*	2.195*	2.259*	2.072*
Mild HIBD vs. mild HIBD + MSCs	5.087^**^	0.247	3.282*	2.009*	3.527^**^	3.698^**^
Severe HIBD vs. severe HIBD + MSCs	3.898^**^	0.00	2.413*	2.588*	2.259*	2.317*

Note: “*” indicates P < 0.05, “**” indicates P < 0.001

### 3.3 Serum levels of IL-6, IL-18, and TNF-α in each group

Analysis of serum IL-6, IL-18, and TNF-α levels was conducted for all groups. Two-way ANOVA followed by multiple comparisons revealed that in both the mild HIBD + MSCs group and the severe HIBD + MSCs group, cytokine concentrations at D0 were significantly different from those at subsequent time points (D3, D7, D11, and D15), with statistical significance (P < 0.001). (See Table 5, Table 7, Table 9)

**Table 5:** Analysis of IL-6 levels in the serum of rats in each group（pg/ml）.

	Control （*n* = 10）	Mild HIBD（*n* = 10）		Severe HIBD（*n* = 10）	Mild HIBD + MSCs（*n* = 10）	Severe HIBD + MSCs（*n* = 10）
D0	19.00 ± 0.61	30.09 ± 0.87		34.84 ± 1.55	29.71 ± 1.26	35.67 ± 1.18
D3	20.68 ± 0.82	28.25 ± 1.40		39.16 ± 1.17	20.57 ± 1.01	29.15 ± 1.04
D7	23.29 ± 0.74	32.15 ± 1.08		39.83 ± 1.62	24.38 ± 1.11	30.51 ± 1.45
D11	21.92 ± 0.97	33.24 ± 1.57		38.81 ± 0.86	26.98 ± 2.21	31.03 ± 0.99
D15	22.68 ± 2.91	29.96 ± 1.33		37.36 ± 1.35	22.96 ± 0.90	28.40 ± 0.81
*F*	13.253	23.738		21.892	65.978	64.942
*P*	＜0.001	＜0.001		＜0.001	＜0.001	＜0.001
Between-group effect			*F*= 1142.842，*P*＜0.001			
Time effect			*F*= 43.523，*P*＜0.001			
Time × Between-group interaction effect			*F*= 33.798，*P*＜0.001			

Note: For multiple comparisons, in the mild HIBD+MSCs group and severe HIBD+MSCs group, D0 vs. D3, D0 vs. D7, D0 vs. D11, and D0 vs. D15 all showed P < 0.001.

Statistical comparisons also showed significant differences in serum cytokine levels between the following groups: control vs. mild HIBD, control vs. severe HIBD, mild HIBD vs. severe HIBD, mild HIBD vs. mild HIBD + MSCs, and severe HIBD vs. severe HIBD + MSCs (P < 0.05). Notably, for comparisons between the mild HIBD and mild HIBD + MSCs groups, as well as the severe HIBD and severe HIBD + MSCs groups at each time point, no significant differences were observed at D0 (P > 0.05), while all other time points exhibited statistically significant differences. (See Table 6, Table 8, Table 10)

**Table 6:** Comparison of IL-6 Levels in Rat Serum.

	T-value					
	ALL	D0	D3	D7	D11	D15
Control vs. mild HIBD	21.694*	32.847*	14.737*	21.389*	19.403*	7.205*
Control vs. severe HIBD	38.313*	29.984*	40.770*	29.418*	41.115*	14.481*
Mild HIBD vs. severe HIBD	16.663*	8.420*	18.864*	12.510*	9.846*	12.331*
Mild HIBD vs. mild HIBD + MSCs	10.083*	0.785	14.050*	15.892*	7.304*	13.792*
Severe HIBD vs. severe HIBD + MSCs	14.056*	1.340	20.157*	13.591*	18.755*	17.981*

Note: “*” indicates P < 0.05

**Table 7:** Analysis of IL-18 levels in the serum of rats in each group（pg/ml）.

	Control （*n* = 10）	Mild HIBD（*n* = 10）		Severe HIBD（*n* = 10）	Mild HIBD + MSCs（*n* = 10）	Severe HIBD + MSCs（*n* = 10）
D0	20.58 ± 1.27	32.34 ± 1.53		41.85 ± 2.15	32.38 ± 1.51	41.75 ± 1.16
D3	22.71 ± 0.99	36.45 ± 1.42		39.59 ± 1.84	28.03 ± 1.33	28.84 ± 1.13
D7	23.53 ± 1.12	30.35 ± 2.59		33.80 ± 2.40	22.66 ± 1.06	30.47 ± 1.42
D11	21.22 ± 1.21	30.73 ± 1.24		37.15 ± 1.83	20.56 ± 0.71	24.32 ± 1.25
D15	23.35 ± 1.00	31.68 ± 1.43		39.77 ± 1.66	25.27 ± 0.84	25.65 ± 1.19
*F*	13.685	20.349		23.798	168.411	312.197
*P*	＜0.001	＜0.001		＜0.001	＜0.001	＜0.001
Between-group effect			*F*= 875.925，*P*＜0.001			
Time effect			*F*= 169.416，*P*＜0.001			
Time × Between-group interaction effect			*F*= 55.972，*P*＜0.001			

Note: In multiple comparisons, within the mild HIBD+MSCs group and severe HIBD+MSCs group, D0 vs. D3, D0 vs. D7, D0 vs. D11, and D0 vs. D15 comparisons all had P < 0.001.

**Table 8:** Comparison of IL-18 Levels in Rat Serum.

	T-value					
	ALL	D0	D3	D7	D11	D15
Control vs. mild HIBD	22.294*	18.718*	25.037*	7.655*	17.332*	15.117*
Control vs. severe HIBD	30.591*	26.965*	25.550*	12.246*	22.970*	26.805*
Mild HIBD vs. severe HIBD	9.948*	11.391*	4.276*	3.087*	9.184*	11.681*
Mild HIBD vs. mild HIBD + MSCs	9.013*	0.052	13.652*	8.701*	22.488*	12.224*
Severe HIBD vs. severe HIBD + MSCs	8.088*	0.119	15.744*	3.769*	18.311*	21.843*

Note: “*” indicates P < 0.05

**Table 9:** Analysis of TNF-α levels in the serum of rats in each group（pg/ml）.

	Control （*n* = 10）	Mild HIBD（*n* = 10）		Severe HIBD（*n* = 10）	Mild HIBD + MSCs（*n* = 10）	Severe HIBD + MSCs（*n* = 10）
D0	44.49 ± 1.47	67.50 ± 2.95		77.00 ± 1.46	67.08 ± 2.80	74.87 ± 3.50
D3	52.59 ± 2.34	62.63 ± 2.17		78.75 ± 1.98	49.20 ± 1.24	55.50 ± 2.22
D7	50.42 ± 1.79	66.16 ± 2.84		73.13 ± 3.17	50.82 ± 1.51	54.78 ± 1.56
D11	44.87 ± 2.00	57.85 ± 2.31		65.62 ± 2.18	45.47 ± 2.72	54.37 ± 1.51
D15	44.04 ± 1.74	64.17 ± 2.34		77.28 ± 2.71	54.09 ± 2.88	64.81 ± 2.13
*F*	43.474	21.701		49.895	125.392	151.325
*P*	＜0.001	＜0.001		＜0.001	＜0.001	＜0.001
Between-group effect			*F*= 1007.972，*P*＜0.001			
Time effect			*F*= 190.221，*P*＜0.001			
Time × Group interaction			*F*= 49.999，*P*＜0.001			

Note: For multiple comparisons, in the mild HIBD+MSCs group and severe HIBD+MSCs group, D0 vs. D3, D0 vs. D7, D0 vs. D11, and D0 vs. D15 all showed P < 0.001.

**Table 10:** Comparison of TNF-α Levels in Rat Serum.

	T-value					
	ALL	D0	D3	D7	D11	D15
Control vs. mild HIBD	20.056*	22.044*	9.937*	14.855*	13.423*	21.838*
Control vs. severe HIBD	28.815*	49.587*	26.949*	19.736*	22.152*	32.668*
Mild HIBD vs. severe HIBD	11.206*	9.118*	17.332*	5.184*	7.737*	11.585*
Mild HIBD vs. mild HIBD + MSCs	8.242*	0.319	16.983*	15.097*	10.963*	8.585*
Severe HIBD vs. severe HIBD + MSCs	9.626*	1.780	24.713*	16.430*	13.422*	11.453*

Note: “*” indicates P < 0.05

## 4 Discussion

Neonatal hypoxic-ischemic encephalopathy (HIBD), a major condition seriously threatening neonatal health, is closely associated with hypoxic-ischemic injury of brain tissue caused by various perinatal insults. This pathological process can result in neurodevelopmental disorders and permanent neurological sequelae, posing a high risk of disability, severely affecting the quality of life of affected infants, and imposing a heavy care burden on families and society (Koehn et al., 2020, Zhang et al., 2021). With ongoing clinical research, increasing evidence suggests a strong association between the pathophysiological progression of HIBD and immune system activation. Immune mediators can activate effector immune cells to release neurotoxic substances, thereby exacerbating central nervous system (CNS) injury (Lan et al., 2021, Lu et al., 2005). Given the high anatomical similarity of rat cerebrovascular structure to that of humans, rats have become ideal animal models for simulating ischemic brain injury and investigating the roles of immune factors in the pathogenesis of HIBD. In a study by Cha Xiaobing et al., elevated levels of multiple immune-related cytokines were detected in the serum of HIBD model rats compared with healthy controls, suggesting a link between immune response and the extent of brain injury (Zha, 2009).

Cytokines, as core molecules in immune regulation, are low-molecular-weight proteins secreted by immune and non-immune cells upon stimulation. By binding to specific receptors, they modulate cellular proliferation, differentiation, and function, playing critical roles in innate immunity, adaptive immune modulation, and tissue repair. In the pathological progression of HIBD, cytokines exhibit bidirectional regulatory roles: pro-inflammatory cytokines (such as IL-6, IL-18, TNF-α) amplify inflammatory cascades and expand neural damage, whereas anti-inflammatory cytokines suppress inflammatory signaling and promote neurological recovery (Huang and Yu, 2019, Yuan, 2020). Notably, cerebrospinal fluid and peripheral blood monocytes are major sources of IL-6, IL-18 (a chemokine subclass), and TNF-α (which selectively targets tumor cells without significant toxicity to normal cells). These cytokines play critical roles in immune modulation and tissue injury by regulating both humoral and cellular immune responses (Wang and Zheng, 2017, Liu et al., 2020).

In this study, cytokine levels were analyzed in HIBD rats with varying severity. Combined with HE staining of brain tissues, clinical observations, and Longa scores, our analysis revealed that both mild and severe HIBD groups showed significantly elevated Longa scores and serum levels of IL-6, IL-18, and TNF-α at all stages compared to the control group (P < 0.05). Moreover, these markers were significantly higher in the severe HIBD group than in the mild group across all stages (P < 0.05), indicating a positive correlation between disease severity and serum cytokine levels. This may be explained by two mechanisms: (1) as the severity of brain injury worsens, amplified inflammation and aggravated tissue damage lead to increased synthesis and secretion of cytokines; (2) increased release of pro-inflammatory cytokines such as IL-6, IL-18, and TNF-α may further aggravate inflammation, impair brain tissue self-repair, and create a vicious cycle of injury amplification and progressive neurological deterioration.

Mesenchymal stem cells (MSCs), a type of fibroblast-like stromal cell with self-renewal and multipotent differentiation capabilities, can be isolated from sources such as bone marrow, umbilical cord blood, placenta, and adipose tissue. Due to their low immunogenicity and immunomodulatory properties, MSCs have shown therapeutic advantages in treating inflammatory diseases. By secreting trophic factors, inhibiting apoptosis, promoting angiogenesis, and regulating the immune microenvironment, MSCs can significantly alleviate neuropathological damage in HIBD model animals (Mishra et al., 2020). Although the precise mechanisms of MSC therapy in HIBD remain unclear, current studies suggest multiple modes of action, including cell replacement, paracrine effects, immunomodulation, anti-apoptotic activity, and improved oxygen supply (Wang and Zou, 2018). Chemotactic factors released following brain injury can guide MSCs to migrate toward injury sites, enabling stage-specific effects such as neuroprotection (within 24 h post-injury) and neural repair (after 24 h) (Parry and Peeples, 2018). Preclinical studies have shown that MSC transplantation 10 days after hypoxia-ischemia promotes regeneration in injured brain regions, improves long-term motor function, and reduces cerebral tissue loss (Braccioli et al., 2017). Animal experiments further demonstrated that MSC transplantation improves cognitive function, sensorimotor ability, and brain histopathology in HIBD rats (Serrenho et al., 2021), with neuroprotective effects potentially associated with apoptosis inhibition and attenuation of inflammation (Ebrahim et al., 2018, Fang et al., 2020).

In this study, rats with varying severities of HIBD received MSC therapy. HE staining and Longa score analysis indicated that both the mild HIBD + MSCs group and the severe HIBD + MSCs group showed improvement in brain tissue damage, with reductions in cell degeneration and nuclear pyknosis. Additionally, Longa scores and serum levels of IL-6, IL-18, and TNF-α significantly decreased at all time points (D3, D7, D11, D15) after MSC treatment compared to pre-treatment levels (D0) (P < 0.05). These findings suggest that MSC therapy can lower serum cytokine levels in HIBD rats, reduce inflammatory responses, improve clinical symptoms, and repair brain injury, thereby providing theoretical support for the clinical application of stem cell transplantation in HIBD treatment.

## 5 Conclusions

In conclusion, serum IL-6, IL-18, and TNF-α levels in HIBD rats are positively correlated with disease severity and may reflect the extent of brain tissue injury, serving as potential indicators for disease assessment. MSC therapy can significantly reduce these cytokine levels, mitigate inflammation, alleviate symptoms, and promote brain tissue repair.

## Conflict of Interest Statement

**All authors confirm that**

- This work is original and unpublished.

- No conflicts of interest exist.

- Data will be shared upon request.

## Funding

This research was funded by 《The Effect of Mesenchymal Stem Cells on the Severity of Hypoxic-Ischemic Brain Damage in Rats by Modulating Serum Cytokines》 of “10.13039/501100004607Guangxi Natural Science Foundation” grant number is “2022GXNSFAA103013”, and The Guangxi Natural Science Foundation was funded by 10.13039/501100010218Department of Science and Technology of Guangxi Zhuang Autonomous Region.

## CRediT authorship contribution statement

**Yuan Tan:** Writing – review & editing, Project administration. **Sifeng Yue:** Writing – original draft, Supervision. **Xinyi Liang:** Data curation. **Shuchun Lü:** Investigation. **Feng Lin:** Writing – original draft, Formal analysis. **Zenghong Huang:** Methodology.

## Data Availability

No new data were created or analyzed in this study. This study does not involve human participants or patient data. All animal procedures were approved by the Institutional Animal Care and Use Committee of Guilin Medical University and were conducted in accordance with the relevant guidelines and regulations.（Animal Ethics Review Number: GLMC202205049）
